# Pre-Diagnosis Pain in Patients With Pancreatic Cancer Signals the Need for Aggressive Symptom Management

**DOI:** 10.1093/oncolo/oyad153

**Published:** 2023-06-07

**Authors:** Terry A McNearney, Biai Dominique Elmir Digbeu, Jacques G Baillargeon, Dennis Ladnier, Lola Rahib, Lynn M Matrisian

**Affiliations:** Scientific and Medical Affairs, Pancreatic Cancer Action Network (PanCAN), Manhattan Beach, CA, USA; Department of Biostatistics, University of Texas Medical Branch, Galveston, TX, USA; Department of Biostatistics, University of Texas Medical Branch, Galveston, TX, USA; Scientific and Medical Affairs, Pancreatic Cancer Action Network (PanCAN), Manhattan Beach, CA, USA; Scientific and Medical Affairs, Pancreatic Cancer Action Network (PanCAN), Manhattan Beach, CA, USA; Scientific and Medical Affairs, Pancreatic Cancer Action Network (PanCAN), Manhattan Beach, CA, USA

**Keywords:** pancreatic cancer pain, health-related quality of life, outcomes, pain morbidity, symptom burden

## Abstract

**Objective:**

This study assessed the impact of pancreatic cancer (PC) pain on associated symptoms, activities, and resource utilization from 2016 to 2020 in an online patient registry.

**Patients and Methods:**

Responses from PC patient volunteers (*N* = 1978) were analyzed from online surveys in a cross-sectional study. Comparisons were performed between PC patient groups reporting, (1) the presence vs. absence of pre-diagnosis PC pain, (2) high (4-8) vs. low (0-3) pain intensity scores on an 11-point numerical rating scale (NRS), and (3) year of PC diagnosis (2010-2020). Descriptive statistics and all bivariate analyses were performed using Chi-square or Fisher’s Exact tests.

**Results:**

PC pain was the most frequently reported pre-diagnosis symptom (62%). Pre-diagnostic PC pain was reported more frequently by women, those with a younger age at diagnosis, and those with PC that spread to the liver and peritoneum. Those with pre-diagnostic PC pain vs. those without reported higher pain intensities (2.64 ± 2.54 vs.1.56 ± 2.01 NRS mean ± SD, respectively, *P* = .0039); increased frequencies of post-diagnosis symptoms of cramping after meals, feelings of indigestion, and weight loss (*P* = .02-.0001); and increased resource utilization in PC pain management: (ER visits *N* = 86 vs. *N* = 6, *P* = .018 and analgesic prescriptions, *P* < .03). The frequency of high pain intensity scores was not decreased over a recent 11-year span.

**Conclusions:**

PC pain continues to be a prominent PC symptom. Patients reporting pre-diagnosis PC pain experience increased GI metastasis, symptoms burden, and are often undertreated. Its mitigation may require novel treatments, more resources dedicated to ongoing pain management and surveillance to improve outcomes.

## Teaser

This study assessed the impact of pancreatic cancer pain on associated symptoms, activities, and resource utilization from 2016 to 2020 in an online patient registry.

**Implications for Practice**

Pain is the most frequently reported pre-diagnosis symptom of pancreatic cancer. Patients with pre-diagnosis pancreatic cancer pain report higher pain intensity, symptoms burden, physical and mood impairment, ER visits, and analgesic prescriptions than those without, signaling the need for immediate and aggressive symptom management for these patients upon diagnosis.

## Introduction

Pancreatic cancer (PC) continues to have a dismal prognosis and high symptom burden from the disease and from side effects of treatment to reduce the tumor.^1,2^ The standard of care (SOC) for PC treatment includes management of PC pain, often requiring opioids.^3-5^

PC pain, described as lower back and/or abdominal pain, is reported to manifest in more advanced stages as a major pre-diagnosis symptom patients report when seeking medical attention and diagnosis.^6-8^ Recently published studies have reported PC pain’s association with patient age, cancer stage, survival, Health-Related Quality of Life (HRQoL), and physical function.^7,9^ Ongoing or undertreated pain is also associated with depression, anxiety, worse physical functioning, and fatigue.^10-12^ Higher pain levels are associated with decreased caloric intake, potentially jeopardizing treatment eligibility and immunocompetence,^7,9,13^ further decreasing patient HRQoL and overall survival.^6,13,14^

The Pancreatic Cancer Action Network (PanCAN) developed several programs to improve patient and caregiver access to care and support under its Patient Services umbrella. In 2016, PanCAN introduced a PC patient registry and survey. Patients were invited to join the patient registry and complete its online PC Survey, allowing the scientific community insight through assessment of symptoms and patient-reported outcomes (PRO) impacted by PC diagnosis and the patient’s experience.^15,16^ Emerging studies have highlighted the importance of cancer-specific PROs, including pain, in prognosis and response to treatment for pancreatic cancer, other cancers, and chronic conditions.^17-22^ Use of standardized, patient-relevant, patient-friendly, disease-specific assessment of the PC experience and PROs in the clinical setting allows improved quality of care by addressing symptoms and conditions linked to HRQol, and in many cases overall survival.

The current study hypothesized that PC patients with pre-diagnosis abdominal and/or back pain, signifying PC pain, also reported higher average pain intensity scores, greater frequencies of associated symptoms, and increased disease and symptom burden.

## Methods

### Data Description

The registry included domains that are important to patients, providers, and researchers. The Health Assessment and 11-point NRS Pain score derived from the Patient-Reported Outcomes Measurement Information System (PROMIS)-29 validated survey.^16,22,23^ Patient Information and PC Experience (from Basics), and Pain Management surveys^16^ were developed as PC-specific surveys. Questions were generated and reviewed by experts in that domain and patients affected by pancreatic cancer. The experts who participated in the review included oncologists, gastroenterologists, scientists, a dietitian, and a radiation oncologist,^16^ similar to survey development protocols reported in other cancer-specific studies.^19^

The data in this study were extracted from the voluntary and self-administered online PanCAN Registry collected between 2016 and 2020. PanCAN’s Patient Registry went online in 2015 and was available to PC patients or anyone interacting with PC patients, such as relatives, friends, Health Care Providers (HCPs), etc. The Patient Registry was used as a tool to collect experiences of pain, symptoms, treatments, medications, clinical, physical, mental, and social activities and management of PC diagnosis and symptoms.^16^ Separate Patient Information, PC Experience, Health Assessment and Pain Management surveys comprised *N* = 108 items of questions or statements used in the current study. The total number of respondents to patient information and PC experience surveys was *N* = 1978, with participation rates of 98.5 and 75%, respectively. Completion of these 2 immediately available surveys at ≥70% was required for access to additional drop-down surveys. This step was used to eliminate possible answer bias and to enrich the data available for identifying disease-state associations with symptoms. Drop-down Health Assessment and Pain Management surveys had lower numbers of respondents, *N* = 168 and *N* = 93, respectively and survey line-item completion rates of 30%-95% and 35%-100%, respectively. Reporting of survey items is identified by capitalization of the first word in the phrase (eg, survey item “feelings of indigestion” is indicated as feelings of indigestion).

Online survey completion trends, from the time (Year, Yr.) of PC diagnosis vs. Yr. of survey completion, are depicted in Supplementary Fig. S1. Decreasing times to survey completion in the later years of PC diagnosis purportedly reflects the increasing participation of recently diagnosed patients accessing the Patient Registry through PanCAN’s Patient Services program.

This non-interventional PRO Registry was approved by an independent institutional review board (IRB) and by Western IRB through the Registry vendor prior to survey launch and yearly thereafter. Survey completion is voluntary as clearly stated in the Registry invitation and informed consent was obtained prior to survey fill-in. The information contained is kept confidential and all identifiers have been removed prior to submission for publication.

### Variables Description

#### Patient Information Survey

Patient demographic items were embedded in the patient information survey and included questions or variables such as age at survey completion, age at PC diagnosis, gender, race, and ethnicity.

#### PC Experience Survey

The PC experience survey included variables such as pre-diagnosis and-post-diagnosis symptoms, characteristics of the PC diagnosed (type, stage, and affected organs), and types of treatment received.

#### Health Assessment Survey

The Health Assessment survey included 5 health domain variables: physical functioning, pain interference, anxiety, depression, and fatigue, comprised of 4 statement items per domain for rating abilities to participate in activities and social roles. Variables were derived from PROMIS (Patient-Reported Outcomes Measurement Information System) using portions of the PROMIS-29 v2.0 survey.^23,24^ Variables reflecting functioning and mood were classified from multiple categories into 2 distinct categories of either “Large” impairment or “Little” impairment by combining possible choices, similar to the categories previously reported^10^

#### Pain Intensity/Rating Item

The pain intensity item embedded in the Health Assessment Survey used an 11-point NRS for pain scoring, based on a scale from 0 = “No pain,” to 10 = “Worst imaginable pain.”^3,25^ The question asked of respondents was, “How would you rate your pain on average?”

#### Pain Management Survey

The drop-down pain management survey included variables such as resource utilization (emergency room (ER) visits and hospitalizations for pain management), doctors/other providers (referred here as HCPs), and pain medications and therapies recommended/prescribed for PC pain management. Also queried were items regarding PC pain characteristics of physical locations, pain types, and descriptors.

### Study Population

**Figure 1.**
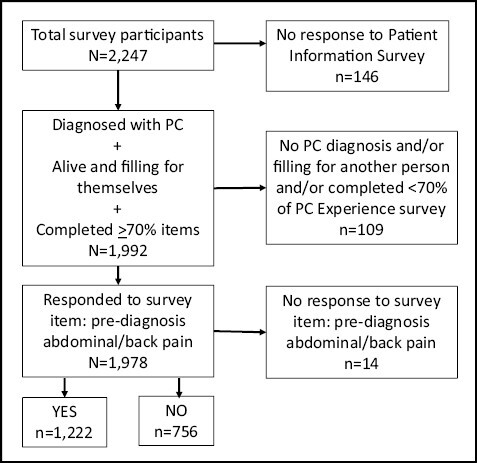
Inclusion and exclusion criteria. The inclusion/exclusion criteria used for this study in the 58 months between 2016 and 2020 is depicted. PC patients who had no responses to the patient information or answered less than 70% of the items in the PC experience surveys were excluded from the study (*N* = 146, 6.5%, top, level 1, right side). Patients who responded “Yes” or “No” to the survey item “Before my diagnosis I had: abdominal and/or back pain,” (pre-diagnosis abdominal/back pain, *N* = 1978, mid-level 3, left side) were included. *N* = number of participants in each population, *n* = number of participants in subcategory.

Survey participants were eligible for this study if: (1) they were diagnosed with PC and completed the Registry for themselves and (2) they responded to a statement item (yes or no) from the PC Experience survey which queried whether participants had pre-diagnosis symptoms of “abdominal and/or back (A/B) pain.” The inclusion/exclusion criteria used for this study between 2016 and 2020 are shown in Fig. 1. Approximately 40 000 PC patients or their representatives accessed PanCAN Patient Services and *N* = 2247 voluntarily joined the online PRO Registry, responding to most or part of the survey items. PC patients who had no or minimal (<70%) responses to the relevant surveys were excluded and those who answered for themselves, *N* = 1992; were included for consideration of study participation. Respondents were further selected those who answered whether they experienced abdominal and/or back pain, indicating PC pain, prior to their diagnosis of pancreatic cancer, *N* = 1978. Hence, the data presented represent single, (nonredundant), patient (not surrogate) responses from PC patient volunteers.

### Statistical Analysis

All the analyses were conducted in SAS 9.4 English version and a *P*-value ≤ .05 was considered significant. Bivariate analysis using Chi-square or Fisher’s exact test for categorical variables and Student’s t test for continuous variables were performed to assess the association of reported pre-diagnosis abdominal and/or back pain symptoms (presence vs. absence) and variables in the surveys related to patient PC experience, health assessment, and pain management.

A sensitivity analysis using the NRS pain variable assessed associations between pain severity and variables in the selected surveys. NRS pain scores were categorized into 2 groups. “Low” NRS pain scores 0-3, no to mild pain, were deemed to have usually manageable pain intensities with little impact on daily activities or mood and “High” NRS pain scores 4-8, moderate to severe pain, were deemed very likely to impact daily activities or mood for most people.^3,10,25^

Additional sensitivity analyses assigned patients to 2 groups, evaluating the associations between the year of PC diagnosis; years prior to 2018 vs. years 2018-2020. Variables compared were days from diagnosis to survey completion, the presence vs. absence of pre-diagnosis PC pain, NRS pain category variables, and PC treatment received to assess response frequency changes in pain intensities between years of survey completion and treatment trends. In addition, a subgroup analysis to compare patients who responded to the survey within 3 months of diagnosis vs. those who responded greater than 19 months after diagnosis was performed.

## Results

### Patient Information

The total number of responses to items from patient information and PC experience surveys, including patient demographics and characteristics of the patients’ PC, is shown in Table 1. The average age at PC diagnosis was 60.5 ± 11.0 years and the survey was completed on average 2 years later. There were approximately equal numbers of male and female respondents and race was predominately White with non-Hispanic ethnicity.

**Table 1.** Characteristics of respondents reporting the presence vs. absence of pre-diagnosis pain. The responses of the PC patient volunteers to the PanCAN patient information and PC experience survey items in the online PanCAN survey (column 1), collected over the 5-year time frame between 2016 and 2020, are shown. Comparisons were generated between PC patients who reported “Yes” or presence of pre-diagnosis abdominal and/or back pain (A/B pain, or pre-diagnosis PC pain) (Column 2) and those who reported “No” or absence of pre-diagnosis abdominal and/or back pain (column 3). The total number of respondents/item are shown (column 4). The *P*-values are shown between comparisons of frequencies (%) of the “Yes” vs. “No” responses (column 5).

Survey items in patient information and PC experience	Pre-diagnosis A/B pain^a^*N* = 1222 *N* (%)	No pre-diagnosis A/B pain^a^*N* = 756 *N* (%)	Total *N* = 1978	*P*-value
Age at time of survey, avg ± SD	61.05±11.08	64.16±10.64	62.24±11.01	**<.0001**
Age at diagnosis, avg ± SD	59.48±11.16	62.23±10.63	60.53±11.04	**<.0001**
Gender				
Female	347 (50.88)	191 (43.02)	538	**.0099**
Male	335 (49.12)	253 (56.98)	588	
Race				
White	559 (87.19)	404 (91.82)	1003	**.0385** ^b^
Black	19 (2.77)	7 (1.59)	26	
Hispanic/Latinx	25 (3.64)	16 (3.64)	41	
Asian	25 (3.64)	10 (2.27)	35	
Other	19 (2.77)	3 (0.68)	22	
Ethnicity				
Non-Hispanic ethnicity	1194 (97.71)	738 (97.62)	1932	.8977
Hispanic ethnicity	28 (2.29)	18 (2.38)	46	
The type of pancreatic cancer, % yes				
Adenocarcinoma (the most common type of pancreatic cancer)	924 (78.84)	562 (77.95)	1486	.4192^b^
Neuroendocrine (also referred to as a PNET or islet cell)	69 (5.89)	54 (7.49)	123	
A type of pancreatic cancer other than those listed above	37 (3.16)	15 (2.08)	52	
The type of pancreatic cancer was not able to be determined	10 (0.85)	5 (0.69)	15	
I don’t know	132 (11.26)	85 (11.79)	217	
Before my diagnosis, I had the following symptoms				
Changes in bowel movements	415 (33.96)	205 (27.12)	620	**.0014**
Digestive problems	676 (55.32)	245 (32.41)	921	**.0001**
Weight loss and/or loss of appetite	672 (54.99)	266 (35.19)	938	**.0001**
Fatigue	565 (46.24)	176 (23.28)	741	**.0001**
Depression	155 (12.68)	41 (5.42)	196	**.0001**
Jaundice (yellowing of skin and whites of eyes)	336 (27.5)	329 (43.52)	665	**.0001**
Dark urine	267 (21.85)	196 (25.93)	463	**.0375**
Itching	193 (15.79)	157 (20.77)	350	**.0049**
Other symptoms not listed above	141 (11.54)	146 (19.31)	287	**.0001**
Before my diagnosis, I had symptoms for				
Less than 1 month	232 (19.25)	187 (35.69)	419	**.0001**
1-4 months	514 (42.66)	204 (38.93)	718	
5-8 months	190 (15.77)	57 (10.88)	247	
8-12 months	93 (7.72)	22 (4.20)	115	
Over one year	139 (11.54)	27 (5.15)	166	
Unknown	37 (3.07)	27 (5.15)	64	
Post-diagnosis symptoms, % Yes				
Cramping after meals	340 (53.63)	121 (34.47)	461	**.0001**
Feelings of indigestion	439 (69.24)	187 (53.28)	626	**.0001**
Weight Loss	455 (71.77)	214 (60.97)	669	**.0005**
Other	78 (12.3)	26 (7.41)	104	**.0166**
Floating or greasy/fatty stools	228 (35.96)	122 (34.76)	350	.7053
Foul-smelling gas and stools	334 (52.68)	180 (51.28)	514	.6737
Frequent stools	203 (32.02)	119 (33.9)	322	.546
Large amounts of gas	404 (63.72)	210 (59.83)	614	.2272
Light-colored, yellow or orange stools	240 (37.85)	134 (38.18)	374	.9206
Loose stools	305 (48.11)	180 (51.28)	485	.3398
Have not experienced any of these symptoms	25 (3.94)	31 (8.83)	56	**.0015**
When I was first diagnosed with pancreatic cancer, my cancer was				
Resectable	37 (3.16)	15 (2.08)	52	.4192^b^
Borderline resectable	924 (78.84)	562 (77.95)	1486	
Locally advanced	132 (11.26)	85 (11.79)	217	
Metastatic	69 (5.89)	54 (7.49)	123	
Unknown	10 (0.85)	5 (0.69)	15	
Currently, my pancreatic cancer is in the following organs,				
Liver	406 (38.48)	210 (31.58)	616	**.0036**
Pancreas	108 (10.24)	46 (6.92)	154	**.0189**
Peritoneum (abdomen)	709 (67.2)	332 (49.92)	1041	**.0001**
Lung	117 (11.09)	85 (12.78)	202	.2885
Lymph nodes	177 (16.78)	89 (13.38)	266	.058
Other	108 (10.24)	45 (6.77)	153	**.0138**
None—currently there is no evidence of disease	57 (5.4)	32 (4.81)	89	.5901
I do not know	181 (17.16)	186 (27.97)	367	**.0001**
Currently on treatment, % yes	611 (64.86)	377 (60.32)	988	.0681
Treatment received, % yes				
Chemotherapy	917 (79.05)	582 (82.09)	1499	.11
Radiation	277 (23.88)	204 (28.77)	481	**.0189**
Surgery	437 (37.67)	427 (60.23)	864	**.0001**
Enrolled in a clinical trial	146 (12.59)	90 (12.69)	236	.9458
Other treatment(s) not listed above	73 (6.29)	56 (7.9)	129	.184
I am not sure	4 (0.34)	1 (0.14)	5	.6555^b^
Did not receive any treatment	133 (11.475)	44 (6.21)	177	.0002

Bold values are statistically significant.

^a^A/B pain, abdominal and/or back pain.

^b^Fisher’s exact test.

PC patients were asked to identify the PC-related pre-diagnosis and post-diagnosis symptoms they experienced from lists of possible choices. The top 3 symptoms reported in the pre-diagnosis period were abdominal and/or back pain (61.8%), weight loss or loss of appetite (47.4%), and digestive problems (46.6%).

### Effect of Pre-Diagnosis Pain on PC Experience

Respondents were placed into 2 groups based on their responses to “Before my diagnosis, I had …. Abdominal and/or back pain”: those with pre-diagnosis pain and those with no pre-diagnosis pain (Table 1). Pre-diagnosis PC associations were not significant for tumor type reported, with similar frequencies for adenocarcinoma (78.5%) and neuroendocrine (6.5%). PC pain was reported in all PC tumor stages at diagnosis (resectable:3.2%; borderline resectable:78.8%; locally advanced: 11.3% and metastatic: 5.9%). Significantly increased frequencies of PC tumor spread to liver (38.5%), peritoneum (67.2%), and other organs (10.2%) associated with the presence of pre-diagnosis pain.

The frequencies of associated pre-diagnosis symptoms were significantly increased in the presence vs. absence of pre-diagnosis PC pain for changes in bowel movements, digestive problems, weight loss or loss of appetite, fatigue, and depression, and significantly decreased for Jaundice and the associated symptoms of dark urine and itching. Post-diagnosis symptom comparisons found significantly increased frequencies in the presence of pre-diagnosis PC pain with weight loss, cramping after meals, and feelings of indigestion, (eg, symptoms that manifest with pain or discomfort in the upper GI region) and significantly decreased frequencies of the item, have not experienced any of these symptoms. Response comparisons were not significant between the presence vs. absence of pre-diagnosis pain and remaining post-diagnosis symptoms related to pronounced GI disruption. The presence of pre-diagnosis PC pain was associated with longer duration of symptoms before PC diagnosis, documented by significantly decreased frequencies for <30 days, while reports of durations between 1 month to over 1 year were consistently higher.

Respondents were also queried on their treatment for PC, of which 63.0% of the total respondents were currently receiving treatment. Treatments over the course of the disease included chemotherapy (80.2%), surgery (46.2%), radiation (25.7%), and enrolled in a clinical trial (12.6%). Patients reporting the presence of pre-diagnosis PC pain had significantly decreased frequencies of radiation and surgery treatments, with no differences for those indicating they received chemotherapy or enrolled in a clinical trial.

### Effect of Pre-Diagnosis Pain on Physical Functioning, Anxiety, Fatigue, and Depression

Patients reporting the presence vs. absence of pre-diagnosis PC pain reported significantly increased frequencies of large impairment in 45% of 20 health assessment activity statements of physical functioning, pain interference, anxiety, fatigue, and depression (Table 2). Pain intensity was increased in those with pre-diagnosis PC pain, with high NRS pain scores significantly associated with the presence (vs. absence) of pre-diagnosis PC pain (34.4% vs. 16.1%, respectively, *P* = .012).

**Table 2.** Effect of pre-diagnosis pain on overall health.

Health assessment survey	Pre-diagnosis A/B pain^a^ *N* = 99	No pre-diagnosis A/B pain^a^ *N* = 69	Total *N* = 168	*P*-value Large impairment
Pain intensity				
NRS pain [0-3]	61 (65.59)	52 (83.87)	113	**.0121**
NRS pain [4-8]	32 (34.41)	10 (16.13)	42	
Physical functioning				
Are you able to do chores such as vacuuming or yard work?				
Always–often	62 (62.63)	58 (84.06)	120	**.0025**
Sometimes—rarely—nnever	37 (37.37)	11 (15.94)	48	
Are you able to go up and down stairs at a normal pace?				
Always–often	78 (79.59)	61 (89.71)	139	.0825
Sometimes—rarely—never	20 (20.41)	7 (10.29)	27	
Are you able to go for a walk of at least 15 minutes?				
Always—often	77 (78.57)	61 (89.71)	138	.0596
Sometimes—rarely—never	21 (21.43)	7 (10.29)	28	
Are you able to run errands and shop?				
Always—often	77 (77.78)	59 (86.76)	136	.1422
Sometimes—rarely—never	22 (22.22)	9 (13.24)	31	
Pain interference				
How much did pain interfere with your day-to-day activities?				
Not at all—a little bit	63 (68.48)	53 (88.33)	116	**.004**
Somewhat—quite a bit—very much	29 (31.52)	7 (11.67)	36	
How much did pain interfere with work around the home?				
Not at all—a little bit	53 (56.99)	46 (73.02)	99	**.0414**
Somewhat—quite a bit—very much	40 (43.01)	17 (26.98)	57	
How much did pain interfere in your ability to participate in social activities?				
Not at all—a little bit	63 (67.74)	51 (82.26)	114	**.0447**
Somewhat—quite a bit—very much	30 (32.26)	11 (17.74)	41	
How much did pain interfere with your household chores?				
Not at all—A little bit	64 (68.82)	54 (88.52)	118	**.0047**
Somewhat—quite a bit—very much	29 (31.18)	7 (11.48)	36	
Anxiety				
I felt fearful				
Never—rarely	53 (56.99)	46 (73.02)	99	**.0414**
Sometimes—often—always	40 (43.01)	17 (26.98)	57	
I found it hard to focus on anything other than my anxiety				
Never—rarely	63 (67.74)	51 (82.26)	114	**.0447**
Sometimes—often—always	30 (32.26)	11 (17.74)	41	
My worries overwhelmed me				
Never—rarely	66 (70.97)	53 (82.81)	119	.0886
Sometimes—often—always	27 (29.03)	11 (17.19)	38	
I felt uneasy				
Never—rarely	46 (49.46)	38 (61.29)	84	.1476
Sometimes—often—always	47 (50.54)	24 (38.71)	71	
Fatigue				
I feel fatigued				
Not at all—a little bit	32 (34.78)	29 (46.77)	61	.1357
Somewhat—quite a bit—very much	60 (65.22)	33 (53.23)	93	
How run-down did you feel on average?				
Not at all—a little bit	41 (43.62)	35 (55.56)	76	.1423
Somewhat—quite a bit—very much	53 (56.38)	28 (44.44)	81	
I have trouble starting things because I am tired				
Not at all—a little bit	47 (51.09)	39 (63.93)	86	.1168
Somewhat—quite a bit—very much	45 (48.91)	22 (36.07)	67	
How fatigued were you on average?				
Not at all—a little bit	34 (36.96)	33 (52.38)	67	.0569
Somewhat—quite a bit—very much	58 (63.04)	30 (47.62)	88	
Depression				
I felt worthless				
Never—rarely	83 (90.22)	59 (95.16)	142	.363^b^
Sometimes—often—always	9 (9.78)	3 (4.84)	12	
I felt helpless				
Never—rarely	64 (69.57)	54 (85.71)	118	**.0205**
Sometimes—often—always	28 (30.43)	9 (14.29)	37	
I felt depressed				
Never—rarely	57 (62.64)	51 (79.69)	108	**.023**
Sometimes—often—always	34 (37.36)	13 (20.31)	47	
I felt hopeless				
Never—rarely	70 (76.92)	53 (86.89)	123	.1255
Sometimes—often—always	21 (23.08)	8 (13.11)	29	

The number (*N*) and frequency of responses (%) to items of the health assessment survey (column 1) from the PC patients is shown based on the presence (column 2) vs. absence (column 3) of pre-diagnosis PC pain. The total numbers of responses to the items are displayed in column 4. Percentages represent column percentages for non-missing data related to each item or question. For the pain intensity item, the NRS pain score responses from *N* = 155 responders were combined into 2 groups, low NRS 0-3 pain scores and high NRS 4-8 pain scores. For the remaining items, 2 score combinations are shown representing little impairment on statement activities (the top descriptors), or large impairment on statement activities (the bottom descriptors). The *P*-values calculated from the patient response differences from the large impact scores, are displayed (column 5). Bold values are statistically significant.

^a^A/B pain, abdominal and/or back pain.

^b^Fisher’s Exact test.

Patients reporting the presence vs. absence of pre-diagnosis PC pain were compared to patient responses to statements of functioning and mood (*N* =20). Combined responses reflected a range of none to little impairment (always or often able to perform task), to large impairment (sometimes, rarely, or never able to perform task). In all statements, the presence of pre-diagnosis pain had a greater frequency of large impairment. A greater percentage of those reporting the absence of pre-diagnosis PC pain were significantly better able to do chores such as vacuuming or yard work compared to those reporting its presence (*P* = .0025), and similar trends were observed with other measures of physical functioning (go up and down stairs at a normal pace, walk for 15 min, run errands). Patients with pre-diagnosis PC pain also reported Large impairment due to pain interference with their day-to-day household in 4/4 statements (*P* = .04 to *P* = .005) and comprised 71%-80.5% of the large impairment group. Patients with pre-diagnosis PC pain reported significantly increased large impairment on 2/4 statements of anxiety (feeling fearful, hard to focus, *P* = .04), with similar trends for the remaining 2 (worries overwhelmed, felt uneasy), and comprised 66%-73% of the large impairment responses. Patients with pre-diagnosis pain reported significantly increased frequencies of large impairment on 2/4 statements of depression, (felt helpless and felt depressed, *P* = .02), with similar trends for the remaining 2 (felt worthless and felt hopeless) and comprised 72%-75% of the large impairment responses. The presence vs. absence of pre-diagnosis PC pain did not differ significantly in large impairment frequencies in 4 statements for fatigue (felt fatigued, How run down? trouble starting things, How fatigued?). However, the large impairment responders of fatigue comprised 64%-67% of patients reporting the presence of pre-diagnosis PC pain.

### Effect of Pre-Diagnostic Pain on Pain Management

Of *N* = 1978 PC patients completing the immediately accessible patient information and PC experience surveys, *N* = 93 answered the pain management survey as depicted in Table 3. PC patients reporting the presence vs. absence of pre-diagnosis PC pain were asked if they are experiencing (or have experienced) pain related to PC to assess their pain management resource utilization, which included emergency room visits, hospitalizations, and HCP visits/contacts for PC pain management. The NRS pain scores from those reporting pre-diagnosis PC pain were significantly higher, (avg ± SD) 2.64 ± 2.56 vs. 1.56 ± 2.01, respectively, *P* = .0039. The total number of patients reporting ≥1 ER visit for pain management was *N* = 45, and a total number of ER visits *N* = 86. Significantly increased frequencies of ER visits were reported in patients also reporting the presence vs. absence of pre-diagnosis PC pain, *N* = 41 vs. *N* = 4, respectively, *P* = .018. Only individuals reporting pre-diagnosis pain reported >2 ER visits. Hospitalizations reported for PC pain management (*N* = 59) showed a similar trend. Patients reporting the presence vs. absence of pre-diagnosis PC pain comprised the majority of hospitalizations, *N* = 56 (95%) vs. *N* = 3 (5%).

**Table 3.** Effect of pre-diagnosis pain on pain management.

Pain management survey items	Pre-diagnosis A/B pain^a^ *N* = 75 *N* (%)	No pre-diagnosis A/B pain^a^ *N* = 18 *N* (%)	Total, *N* = 93	*P*-value
Pain rating score, NRS pain, Avg ± SD^	2.64±2.56	1.56±2.01	2.21±2.41	**.0039**
Visited the emergency room because of the pain related to your PC: yes, %	41 (54.67)	4 (22.22)	45	**.0177** ^b^
Number of times visited the emergency room because of pain related to your PC: yes, %				
1	16 (38.1)	2 (50)	18	1.0000^b^
2	17 (40.48)	2 (50)	19	
3	6 (14.29)	0 (0)	6	
4	3 (7.14)	0 (0)	3	
Hospitalized because of pain related to your pancreatic cancer: yes, %	29 (38.67)	3 (16.67)	32	.1003^b^
Number of times hospitalized because of pain related to your pancreatic cancer				
1	10 (33.33)	3 (100)	13	.1749^b^
2	15 (50)	0 (0)	15	
3	4 (13.33)	0 (0)	4	
4	1 (3.33)	0 (0)	1	
Discussed pain with my doctor (or other healthcare professional)	31 (93.94)	14 (100)	45	1.0000
My doctor has recommended and/or prescribed pain medication for my pain:	68 (98.55)	15 (83.33)	83	**.0267** ^b^
The type(s) of pain medication my doctor (or healthcare professional) has recommended/prescribed is/are				
Opioid [fentanyl (duragesic), oxycodone (Percocet), morphine (MS contin)]	61 (89.71)	12 (85.71)	73	.6469^b^
Over the counter medication	9 (13.24)	3 (21.43)	12	.4216^b^
Prescription strength non-opioids (acetaminophen, ibuprofen, aspirin)	13 (19.12)	5 (35.71)	18	.1771^b^
Anti-depressant for tingling or burning pain [gabapentin (neurontin), amitriptyline (elavil)]	7 (10.29)	4 (28.57)	11	.0874^b^
Another type of medication not listed above	10 (14.71)	2 (14.29)	12	1.0000^b^
No recommendations were made by the doctor/healthcare professional	21 (32.31)	4 (28.57)	25	1.0000^b^
Other pain treatment modalities recommended/prescribed by my HCP, (*N* = 14 modalities)	134	28	NA	ND

Patient numbers (*N*) and frequencies (%) who indicated they are experiencing or have experienced pain related to pancreatic cancer from the pain management survey (column 1), based on PC patients reporting the presence (column 2) or absence (column 3) of pre-diagnosis PC pain. The total numbers of responses for each item are shown (column 4). Percentages values represent column percentages for non-missing data related to each item/question. The *P*-values for each item comparison are shown (column 5). Other pain treatment modalities recommended/prescribed by my HCP (*N* = 14): Herbal remedies, hot or cold packs, exercise, changing positions, (such as laying down or elevating your legs), physical therapy, massage, acupuncture, rest, guided imagery, relaxation techniques (hypnosis, biofeedback, and meditation), creative techniques, (art or music therapy), chiropractic treatment, osteopathic treatment, another recommendation not listed above. Combined number of responses from patients reporting the presence vs. absence of pre-diagnosis PC pain regarding the 14 additional treatment options were *N* = 134 vs. *N* = 28, respectively (not shown). Bold values are statistically significant.

^a^A/B pain, abdominal and/or back pain.

^b^Fisher’s Exact test.

Most patients reported discussing pain with their HCP, who recommended/prescribed medications for pain control (prescribed, over the counter analgesics and nonpharmacologic therapies), with a slightly higher frequency in those reporting the presence vs. absence of pre-diagnosis PC pain (98.6% vs. 83.3%, respectively, *P* = .027). Those reporting the presence of pre-diagnosis PC pain comprised 67%-100% of recommended/prescribed pain medications queried. Frequencies of prescriptions/recommendations reported for opioids, nonopioids, and over the counter medications were the same for both groups. Responses to additional therapeutic nonpharmacologic options were also queried. Responses of the presence of pre-diagnosis PC pain who provided NRS pain scores found highest frequencies of pain located in the upper abdominal and lower back regions, chronic >acute pain types, and all 6 pain descriptors used by PC patients, shown in Supplementary Table S1.

### Effects of Pain Intensity and PC Experience

Of *N* = 1978 PC patients completing the immediately accessible patient information and PC experience surveys, *N* = 155 answered the NRS pain rating question in the health assessment survey as depicted in Supplementary Table S2. PC patient pain rating responses were within the numeric range of 0-8 out of a possible rating scale from 0 to 10, with high NRS 4-8 pain scores reported by 27.1% and low NRS 0-3 by 72.9% of respondents. The frequency of NRS pain scores = 0, or patients reporting no pain, was 36.8%, with 36.1% reporting NRS pain scores of 1-3. Both presence vs. absence of pre-diagnosis PC pain groups contained responses of NRS pain = 0 at a frequency of 31.2% vs. 50%, respectively.

Responses from patient information and PC experience surveys by PC patients assigned to high (NRS 4-8, *N* = 42) vs. low (NRS 0-3, *N* = 113) pain score groups, reported post-diagnosis at survey completion, are compared in Supplementary Table S3. The high vs. low NRS pain group had significantly lower average age at survey completion, was more likely to indicate lymph node metastasis, and reported decreased frequencies of no evidence of disease and having received surgery for PC treatment. The high-pain group had significantly increased frequencies of patients reporting pre-diagnosis abdominal and/or back pain, digestive problems, and fatigue. There were no differences in post-diagnosis symptoms between the high- and low-pain groups. There was also no difference in the percent of individuals with adenocarcinoma vs. neuroendocrine types of pancreatic cancer who reported pre-diagnosis pain, or reported high vs low NRS pain scores, despite the significant differences in the survival rates between the 2 pancreatic cancer histologies (data not shown).

To determine if the delay in survey completion (an average of 2 years after diagnosis) resulted in bias in recall of pain intensity, the percent of individuals reporting high vs. low pain and completing the survey within 2 months vs. greater than 18 months after diagnosis was compared (Supplementary Table S4). Although there is a trend for a somewhat higher percentage of individuals who reported high vs. low pain within those who filled out the survey shortly after diagnosis, this was not statistically significant and suggests there is no bias related to the time after diagnosis of survey completion.

The responses of PC patients assigned to high vs. low pain groups in the health assessment survey were compared for their impairment in physical functioning, pain interference with normal activities, anxiety, fatigue, and depression (Table 4). Average ± SD of NRS pain scores between high and low NRS pain groups were 6.28 ± 1.05 vs.1.16 ± 1.39, respectively, *P* = .0001. High NRS pain scores associated with significantly increased frequencies of large impairment: of physical functioning, (4/4 questions, *P* = .001); pain interference, (4/4 questions, *P* = .0001); feelings of anxiety, (4/4 statements, *P* = .03-.003); activities related to fatigue, (4/4 statements, *P* = .001) and feelings of depression, (felt depressed, *P* = .02, with the remaining 3 statements trending in the same direction).

**Table 4.** Effect of pain intensity on overall health.

Health assessment survey	Pain NRS 4-8 *N* = 42 *N* (%)	Pain NRS 0-3 *N* = 113 *N* (%)	Total *N* = 155	*P*-value Large impairment
NRS pain score, Avg ± SD	6.28±1.05	1.16±1.39	2.21±2.41	**<.001**
Physical functioning				
Are you able to do chores such as vacuuming or yard work?				
Always—often	19 (45.24)	94 (83.19)	113	**.001**
Sometimes—rarely—never	23 (54.76)	19 (16.81)	42	
Are you able to go up and down stairs at a normal pace?				
Always—often	26 (65)	103 (91.15)	129	**.0001**
Sometimes—rarely—never	14 (35)	10 (8.85)	24	
Are you able to go for a walk of at least 15 min?				
Always—often	24 (60)	104 (92.04)	128	**.0001**
Sometimes—rarely—never	16 (40)	9 (7.96)	25	
Are you able to run errands and shop?				
Always—Often	25 (60.98)	101 (89.38)	126	**.0001**
Sometimes—rarely—never	16 (39.02)	12 (10.62)	28	
Pain interference				
How much did pain interfere with your day-to-day activities?				
Not at all—a little bit	12 (30)	104 (93.69)	116	**.0001**
Somewhat—quite a bit—very much	28 (70)	7 (6.31)	35	
How much did pain interfere with work around the home?				
Not at all—a little bit	10 (25)	107 (94.69)	117	**.0001** ^a^
Somewhat—quite a bit—very much	30 (75)	6 (5.31)	36	
How much did pain interfere in your ability to participate in social activities?				
Not at all—a little bit	13 (31.71)	104 (93.69)	117	**.0001**
Somewhat—quite a bit—very much	28 (68.29)	7 (6.31)	35	
How much did pain interfere with your household chores?				
Not at all—a little bit	11 (27.5)	107 (94.69)	118	**.0001** ^a^
Somewhat—quite a bit—very much	29 (72.5)	6 (5.31)	35	
Anxiety				
I felt fearful				
Never—rarely	19 (46.34)	78 (70.27)	97	**.0064**
Sometimes—often–always	22 (53.66)	33 (29.73)	55	
I found it hard to focus on anything other than my anxiety				
Never—rarely	22 (55)	88 (79.28)	110	**.0031**
Sometimes—often—always	18 (45)	23 (20.72)	41	
My worries overwhelmed me				
Never—rarely	24 (58.54)	91 (81.25)	115	**.004**
Sometimes—often—always	17 (41.46)	21 (18.75)	38	
I felt uneasy				
Never—rarely	16 (40)	67 (60.36)	83	**.0265**
Sometimes—often—always	24 (60)	44 (39.64)	68	
Fatigue				
I feel fatigued				
Not at all—a little bit	4 (9.76)	56 (50.91)	60	**.0001** ^a^
Somewhat—quite a bit—very much	37 (90.24)	54 (49.09)	91	
How run-down did you feel on average?				
Not at all—a little bit	7 (17.07)	68 (60.18)	75	**.0001** ^a^
Somewhat—quite a bit—very much	34 (82.93)	45 (39.82)	79	
I have trouble starting things because I am tired				
Not at all—A little bit	11 (26.19)	74 (68.52)	85	**.0001**
Somewhat—quite a bit—very much	31 (73.81)	34 (31.48)	65	
How fatigued were you on average?				
Not at all—a little bit	7 (16.67)	59 (53.64)	66	**.0001** ^a^
Somewhat—quite a bit—very much	35 (83.33)	51 (46.36)	86	
Depression				
I felt worthless				
Never—rarely	35 (85.37)	104 (94.55)	139	.0636
Sometimes—often—Always	6 (14.63)	6 (5.45)	12	
I felt helpless				
Never—rarely	28 (66.67)	88 (80)	116	.0838
Sometimes—often—always	14 (33.33)	22 (20)	36	
I felt depressed				
Never—rarely	23 (54.76)	82 (74.55)	105	**.0183**
Sometimes—often—always	19 (45.24)	28 (25.45)	47	
I felt hopeless				
Never—rarely	29 (70.73)	91 (84.26)	120	.0625
Sometimes—often—always	12 (29.27)	17 (15.74)	29	

The responses to items of the Health health Assessment assessment survey (column 1) from the PC patients with high (column 2) or low (column 3) NRS pain scores are shown. The total number of responses to items is displayed in column 4. Percentages represent column percentages for non-missing data related to each item/question. Two score combinations are shown under each statement to assess pain NRS scores, either little impairment on statement activities ( top descriptors), or large impairment on statement activities (bottom descriptors). The *P*-values calculated from the patient response differences from the large impairment groups are displayed (column 5). Bold values are statistically significant.

^a^Fisher’s Exact test.

### Frequency of Reported Pain Scores by Year of PC Diagnosis

To determine if trends in PC pain changed over the past decade, Table 5 shows high vs. low NRS pain scores based on the year of PC diagnosis. Of patients diagnosed in years prior to 2018 (*N* = 30), 23.8% reported high NRS pain scores at survey completion. Patients diagnosed with PC in years. 2018-2020 (*N*= 1 2), 41.4% reported high NRS pain scores, *P* = .055, notably not significantly reduced over the 11-year span of PC diagnoses. Patients diagnosed with PC prior to 2018 vs. 2018-2020 had decreased frequencies of did not receive any treatment and increased frequencies of chemotherapy, radiation, and surgery as treatments, shown in Supplementary Table S5.

**Table 5.** Pain scores by year of PC diagnosis.

Years of PC diagnosis	High pain (NRS 4-8) *N* = 42 *N* (%)	Low pain (NRS 0-3) *N* = 113 *N* (%)	Total *N* = 155	*P*-value
PC diagnosis prior to 2018	30 (23.8)	96 (76.2)	126	.0549
PC diagnosis in 2018-2020	12 (41.4)	17 (58.6)	29	

PC patient numbers (*N*) and frequencies (%) of high (column 2) and low (column 3) NRS pain score based on year of PC diagnosis divided into groups of diagnosis prior to 2018 (2010-2017) and diagnosis 2018-2020 (column 1). Percentages represent column percentages for non-missing data related to each item/question. The total number of responses for the years of PC diagnosis are shown in column 4 and the *P*-value obtained by comparing the high pain scores is shown in column 5.

## Discussion

This cross-sectional study presented unique single-time point responses to an online survey over a 5-year period between 2016 and 2020 from close to 2000 PC patients visiting PanCAN’s Patient Services and voluntarily completing the Patient Registry survey. The most frequently reported pre-diagnosis symptom was PC pain, which aligned with previous reports.^2,26,27^ The initial assessment comparing the presence of pre-diagnosis PC pain showed significantly increased responses to symptoms reflecting GI dysfunction and GI-based local and distant tumor spread. These survey responses support local pancreatic tumor invasion of the local or communicating neural sheaths and ensuing disruption or ongoing release of neurotransmitters (eg, 5-HT, BDNF, CGRP, and NE) to produce or enhance GI tract pathology.^28-31^ Systemically, significantly increased associations of pre-diagnosis PC pain and impairment of functioning and mood were reported, consistent with previous reports.^7,9,32^

The second assessment of NRS pain scores found substantial impairment of daily physical activities, mood statements, and increased frequencies of pre-diagnosis PC pain with high (vs. low) NRS pain scores. Previous reports of poor outcomes have been associated with increased pain levels, depression and fatigue, decreased physical and social functioning,^5,7,32,33^ and increased anxiety.^11,12^

The third comparison of resource utilization between the presence vs. absence of pre-diagnosis PC pain found increased ER and hospital visits and increased HCP recommendations for PC pain management. Increased frequencies of HCPs recommending/prescribing analgesics, including opioids, for most of the reported PC pain were noted, indicating HCP responsiveness to PC patient pain complaints.

Two time periods queried (pre-and post-diagnosis symptoms) of the PC patients’ clinical courses showed that PC pain levels can be dynamic and most likely responsive to intervention. Some reporting the presence of pre-diagnosis PC pain also reported post-diagnosis NRS pain scores = 0 and alternatively, some patients reporting the absence of pre-diagnosis PC pain noted post-diagnosis NRS pain scores >0. NRS = 0 pain scores, or no pain, were reported in 37% of responders at post-diagnosis survey completion, in agreement with a previous study.^34^

PC pain is reported with poor outcomes, as its onset and severe intensity are reported in advanced disease.^6^ The presence of pre-diagnosis PC pain was associated with increased frequencies of GI symptoms, all reported cancer stages (highest in borderline resectable), and spread to GI tissues. It is also associated with increased frequencies of higher NRS pain scores, large impairment of daily and social activities, and increased resource utilization in ongoing PC patient management. The presence of pre-diagnosis PC pain and high NRS pain scores appear to act as sufficient and also possibly additive or synergistic contributors to reported large impairment of activities and mood. The absence of pre-diagnosis PC pain was associated with significantly increased frequencies of jaundice, symptoms related to biliary obstruction, and earlier PC diagnosis.^9,14^

This study had several limitations. This was a cross-sectional study and self-administered, limiting patient responses to one-time point, post-diagnosis, with biases intrinsic to voluntary survey completion. Large numbers of responses obtained from the immediately available patient information and PC experience surveys were reduced to 5%-9% in the drop-down surveys of health assessment and pain management, precluding further analyses with associated variables and raising concerns of responder bias. Small patient participation in survey completion has been reported for voluntary surveys^.35^ Also, response interpretation of the pain-rating item was limited, as patient orientation to pain type, anchoring, location, time frame, etc. were unavailable.

These survey responses of pre-diagnosis PC pain signals strongly support a multitude of previous article positions on the need for effective, ongoing symptom and resource management, regardless of PC prognosis. It is notable that the frequencies of high NRS pain scores did not decrease over a recent 11-year span available for study, possibly due to diagnosis and treatment confounders, or at least in part due to undertreated pain. Symptoms related to tissue edema, ischemia, and neuropathic injury will probably require additional, novel therapies to current analgesic SOC, including novel modulators of tissue edema and mechanoreceptors, (eg,^36-38^) and mitigation of neurogenic and inflammatory contributors to neuropathic pain.^39-41^ Multidisciplinary treatment groups should strongly consider increased participation of pain medicine/palliative care specialties soon after PC diagnosis to optimize pain management in the presence of pre-diagnosis PC pain that is ongoing, and/or NRS pain scores ≥4.^42-46^ Recent articles support timely involvement of palliative care specialties for management of weight and symptoms to decrease overall resource utilization.^47-49^ Their earlier involvement in patient care may require corporate or government policy changes to provide consistent evolving management of symptoms to improve HRQoL and overall health and survival in PC.^10,45,50-53^ Finally, the use of patient surveys querying symptoms and treatments independent of clinical studies offers adjunctive instruments to spot trends, consequences, contributors, and mitigators of symptom intensities. Future use of PanCAN survey responses could further assess the “real world” therapeutic potential of SOC, off-label, experimental, and integrative treatments, such as radiation therapy, chemotherapy, surgical excision or ablation, and interventional analgesic delivery.^14,54,55^

## Conclusion

PC pain is a prominent PC symptom in all PC stages, dynamic in intensity and causation, often reported in the pre-diagnosis interval, and often undertreated. Pain is a modifiable risk factor for poor outcomes and worse prognosis. Treatment of downstream consequences of undermanaged PC pain is less desirable and inefficient. Its successful mitigation will require early dedicated, ongoing pain management. A better alignment of tumor reduction with patient symptom reduction will contribute to improved functioning, HRQoL, and overall survival.

## Supplementary Material

oyad153_suppl_Supplementary_Tables

## Data Availability

The data underlying this article will be shared on reasonable request to the corresponding author.
