# Supplementary material for: Pre-Diagnosis Pain in Patients With Pancreatic Cancer Signals the Need for Aggressive Symptom Management
Source: Oncologist. 2023 Jun 7;28(12):e1185–97. doi: 10.1093/oncolo/oyad153 (PMC10712702; doi:10.1093/oncolo/oyad153)
Supplement: oyad153_suppl_Supplementary_Tables [file oyad153_suppl_supplementary_tables.docx]

**Supplemental Tables**

| **Supplemental Table s1** | **Pre-diagnosis A/B pain, N (%),**  **Total N=93** | **No Pre-diagnosis A/B pain, N (%),** **Total N=62** | **Total N=155** | **p-value** |
| --- | --- | --- | --- | --- |
| **Body location, type and descriptors of PC patient reported pain** | | | | |
| ***Experiencing (or have experienced) Mid-back pain*** | **37 (48.68)** | **6 (31.58)** | **43** | **0.180** |
| My mid-back pain is/was acute pain | 16 (44.44) | 2 (33.33) | 18 | 0.611 |
| My mid-back pain is/was chronic pain | 24 (66.67) | 4 (66.67) | 28 | 1.000 |
| My mid-back pain is/was breakthrough pain | 8 (22.22) | 4 (66.67) | 12 | 0.028 |
| My mid-back pain could best be described as throbbing | 2 (33.33) | 2 (33.33) | 4 | 1.00 |
| My mid-back pain could best be described as sharp | 16 (44.44) | 2 (33.33) | 18 | 0.611 |
| My mid-back pain best be described as tingling/burning | 7 (19.44) | 0 (0) | 7 | 0.237 |
| My mid-back pain could best be described as cramp-like | 5 (13.89) | 1 (16.67) | 6 | 0.857 |
| My mid-back pain could best be described as dull | 23 (63.89) | 5 (83.33) | 28 | 0.350 |
| ***Experiencing (or have experienced) pain in the lower back*** | **45 (59.21)** | **5 (26.32)** | **50** | **0.010** |
| My lower back pain is/was: Acute pain | 14 (33.33) | 4 (80) | 18 | 0.042 |
| My lower back pain is/was: Chronic pain | 28 (66.67) | 3 (60) | 31 | 0.766 |
| My lower back pain is/was: Breakthrough pain | 10 (23.81) | 3 (60) | 20 | 0.087 |
| My lower back pain could best be described as throbbing | 18 (42.86) | 2 (50) | 20 | 1.000 |
| My lower back pain could best be described as sharp | 16 (38.1) | 1 (25) | 17 | 0.604 |
| My lower back pain best be described as tingling/burning | 5 (11.9) | 0 (0) | 5 | 0.465 |
| My lower back pain could best be described as cramp-like | 6 (14.29) | 0 (0) | 6 | 0.418 |
| My lower back pain could best be described as dull | 22 (52.38) | 3 (75) | 25 | 0.614 |
| ***Experiencing (have experienced) upper abdominal pain?*** | **61 (80.26)** | **12 (63.16)** | **73** | **0.114** |
| Upper abdominal pain is/was: Acute pain | 25 (43.86) | 4 (36.36) | 29 | 0.645 |
| Upper abdominal pain is/was: Chronic pain | 42 (73.68) | 6 ( 54.55) | 48 | 0.202 |
| Upper abdominal pain is/was: Breakthrough pain | 18 (31.58) | 5 (55.5) | 23 | 0.373 |
| My upper abdominal pain is best described as throbbing | 16 (28.57) | 3 (25) | 39 | 1.000 |
| My upper abdominal pain is best described as sharp | 42 (73.68) | 6 (54.55) | 48 | 0.202 |
| My upper abdominal pain best described as tingling/burning | 6 (10.71) | 1 (8.33) | 7 | 1.000 |
| My upper abdominal pain is best described as cramp-like | 28 (50) | 8 (66.67) | 36 | 0.325 |
| My upper abdominal pain is best described as dull | 31 (55.36) | 7 (58.33) | 38 | 1.0 |
| ***Experiencing (or have experienced) lower abdominal pain?*** | **44 (57.89)** | **12 (63.16)** | **56** | **0.676** |
| Lower abdominal pain is was: Acute | 19 (45.24) | 8 (72.73) | 27 | 0.175 |
| Lower abdominal pain is was: Chronic | 28 (66.67) | 7 (63.64) | 35 | 1.0 |
| Lower abdominal pain is was: Breakthrough | 16 (38.1) | 5 (45.45) | 21 | 0.736 |
| My lower abdominal pain is best described as throbbing | 11 (25.58) | 3 (25) | 14 | 0.105 |
| My lower abdominal pain is best described as sharp | 23 (53.49) | 6 (50) | 29 | 1.000 |
| My lower abdominal pain best described as tingling/burning | 6 (13.95) | 2 (16.67) | 8 | 1.000 |
| My lower abdominal pain is best described as cramp-like | 6 (14.29) | 0 | 6 | 1.000 |
| My lower abdominal pain is best described as dull | 22 (52.38) | 3 (75) | 25 | 0.614 |
| ***Experiencing (experienced) pain in other body areas*** | ***7 (9.21)*** | ***4 (21.05)*** | ***11*** | ***0.222*** |
| Pain in other body area(s) is/was Acute Pain | *3 (42.86)* | *1 (8.33)* | *4* | *0.556* |
| Pain in other body area(s) is was Chronic Pain | *6 (85.71)* | *3 (75)* | *9* | *0.658* |
| Pain in other body area(s)is/was Breakthrough pain | *1 (14.29)* | *1 (8.33)* | *2* | *0.758* |
| Pain in other body area(s) best described as: Throbbing | *4 (57.14)* | *1 (25)* | *5* | *1.000* |
| Pain in other body area(s) best described as: Sharp | *5 (71.43)* | *2 (50)* | *7* | *0.576* |
| Pain in other body area(s) best described as: Tingling/burn | *4 (57.14)* | *2 (50)* | *6* | *1.000* |
| Pain in other body area(s) best described as: Cramp-like | *4 (57.14)* | *1 (25)* | *5* | *0.546* |
| Pain in other body area(s) best described as: Dull | *4 (57.14)* | *0* | *4* | *0.194* |

**Supplemental Table s1. Comparison of pain location, type and descriptor responses between patients reporting the presence vs. absence of pre-diagnosis PC pain.** *Body locations:* mid back, lower back, upper abdomen, lower abdomen, other body areas. *Types of pain experienced:* Acute: begins suddenly and lasting for short periods, ranging as mild to severe in intensity; Chronic: Chronic pain constant or recurs frequently and lasts for a long period of time; it can range from mild to severe in intensity; Breakthrough: common in patients with chronic pain it occurs suddenly and is usually short-lived; it can range from moderate to severe in intensity*. Descriptors of pain:* throbbing, sharp, tingling/burning, cramp-like and dull. NOTE: Term definitions for type of pain experienced and pain descriptors were not available to PC patient respondents**.**

The greatest frequencies of responses are Lower back and Upper abdominal areas. Response rates to body areas range from 78-11%. The PC patients who reported the presence of pre-diagnosis Abdominal and/or back pain comprised the majority of responses to this section of the Pain Management survey. The more frequent responses of pain type were to Chronic, then Acute and to pain descriptors: Sharp and Dull. The lowest frequency of responses to pain descriptors was Tingling and Burning. The frequencies of responses were low for these sets of items. Note higher frequencies for Regions of Upper GI and Lower back, very low frequencies for pain in other body locations (15%) indicating the prominence of PC pain to the responders and increased frequencies of pain descriptors, Sharp and Dull. Almost all pain descriptors are located in all body regions. Patients often responded Yes with > 1 pain areas, types and descriptors.

**Supplemental Table s2**

No pain

Worst imaginable pain

| **NRS** | **N** | **%** |
| --- | --- | --- |
| 0 | 57 | 36.77 |
| 1 | 22 | 14.19 |
| 2 | 18 | 11.61 |
| 3 | 16 | 10.32 |
| 4 | 12 | 7.74 |
| 5 | 8 | 5.16 |
| 6 | 10 | 6.45 |
| 7 | 7 | 4.52 |
| 8 | 5 | 3.23 |
| 9 | 0 | 0 |
| 10 | 0 | 0 |

**Supplemental Table s2. Pain intensity/rating.** The pain score distribution from the Pain Rating item “Pain intensity: How would you rate your pain on average?” on an 11-point numerical rating scale (NRS), ranging from “0” indicating “no pain” to “10” indicating the “worst imaginable pain” is displayed. The PC patient responses ranged from NRS=0 indicating none or no pain, to NRS=8 indicating a score that corresponds to severe pain intensity. The numbers (N) of patient responses and the frequencies of those responses (%) are also displayed. Out of the N=1978 PC patients who completed the immediately accessible Patient information and PC Experience surveys, N=155 have answered the pain rating item in the drop-down Health Assessment survey.

| **Supplemental Table s3. Items from Patient Information and PC Experience Surveys** | **Pain NRS 4-8 N=42** | **Pain NRS 0-3 N=113** | **Total N=155** | **p-value** |
| --- | --- | --- | --- | --- |
| Age at time of survey, Avg ± SD | 57.52 +12.07 | 62.05 +10.99 | 2.21 +2.41 | **0.0285** |
| Age at diagnosis, Avg ± SD | 55.48 +11.99 | 59.17 +12.11 |  | 0.0947 |
| Gender |  |  |  |  |
| Female | 16 (42.11) | 50 (48.08) | 66 | 0.5276 |
| Male | 22 (57.89) | 54 (51.92) | 76 |  |
| Race |  |  |  |  |
| White | 35 (92.11) | 99 (95.19) | 134 | 0.3666* |
| Black | 1 (2.63) | 1 (0.96) | 2 |  |
| Hispanic/Latinx | 2 (5.26) | 2 (1.92) | 4 |  |
| Asian | 0 (0)ABO | 2 (1.92) | 2 |  |
| Other | 0 | 0 (0) | 0 |  |
| Ethnicity |  |  |  |  |
| Non-Hispanic | 40 (95.24) | 110 (97.35) | 150 | 0.6129* |
| Hispanic | 2 (4.76) | 3 (2.65) | 5 |  |
| Before my (PC) diagnosis, I had the following symptoms |  |  |  |  |
| Abdominal and/or back pain | 32 (76.19) | 61 (53.98) | 93 | **0.0121** |
| Changes in bowel movements | 12 (28.57) | 30 (26.55) | 42 | 0.8012 |
| Digestive problems | 27 (64.29) | 46 (40.71) | 73 | **0.009** |
| Weight loss and/or loss of appetite | 22 (52.38) | 47 (41.59) | 69 | 0.2297 |
| Fatigue | 20 (47.62) | 32 (28.32) | 52 | **0.0237** |
| Depression | 8 (19.05) | 12 (10.62) | 20 | 0.1642 |
| Jaundice (yellowing of skin and whites of eyes) | 13 (30.95) | 39 (34.51) | 52 | 0.6764 |
| Dark Urine | 12 (28.57) | 27 (23.89) | 39 | 0.5509 |
| Itching | 7 (16.67) | 19 (16.81) | 26 | 0.9826 |
| Other symptoms not listed above** | 8 (19.05) | 13 (11.5) | 21 | 0.2226 |
| Before my diagnosis, I had symptoms for |  |  |  |  |
| Less than one month | 4 (10.26) | 25 (25.51) | 29 | 0.2158* |
| 1-4 months | 19 (48.72) | 40 (40.82) | 59 |  |
| 5-8 months | 6 (15.38) | 14 (14.29) | 20 |  |
| 8-12 months | 3 (7.69) | 6 (6.12) | 9 |  |
| Over one year | 7 (17.95) | 9 (9.18) | 16 |  |
| Unknown | 0 (0) | 4 (4.08) | 4 |  |
| The type of pancreatic cancer I was diagnosed with |  |  |  |  |
| A type of pancreatic cancer other than those listed below | 0 (0) | 5 (4.59) | 5 | 0.2625* |
| Adenocarcinoma (most common type of pancreatic cancer) | 38 (90.48) | 88 (80.73) | 126 |  |
| I don't know | 3 (7.14) | 6 (5.5) | 9 |  |
| Neuroendocrine (also referred to as a PNET or islet cell) | 1 (2.38) | 10 (9.17) | 11 |  |
| The type of pancreatic cancer was not able to be determined | 0 (0) | 0 (0) | 0 |  |
| Post-diagnosis symptoms, % Yes |  |  |  |  |
| Cramping after meals | 11 (55) | 21 (45.65) | 32 | 0.485 |
| Feelings of indigestion | 15 (75) | 31 (67.39) | 46 | 0.5365 |
| Weight Loss | 15 (75) | 36 (78.26) | 51 | 0.7714 |
| Other | 4 (10.26) | 10 (9.52) | 14 | 1.0000* |
| Floating or greasy/fatty stools | 7 (35) | 19 (41.3) | 26 | 0.63 |
| Foul smelling gas and stools | 13 (65) | 28 (60.87) | 41 | 0.7506 |
| Frequent stools | 8 (40) | 17 (36.96) | 25 | 0.8148 |
| Large amounts of gas | 15 (75) | 32 (69.57) | 47 | 0.654 |
| Light-colored, yellow or orange stools | 8 (40) | 30 (65.22) | 38 | 0.0568 |
| Loose stools | 12 (60) | 26 (56.52) | 38 | 0.7927 |
| Have not experienced any of these symptoms | 3 (15) | 2 (4.35) | 5 | 0.1587* |
| When I was first diagnosed with pancreatic cancer, my cancer was | |  |  |  |
| Resectable | 10 (23.81) | 29 (25.89) | 39 | 0.7514* |
| Borderline resectable | 9 (21.43) | 34 (30.36) | 43 |  |
| Locally advanced | 7 (16.67) | 14 (12.50) | 21 |  |
| Metastatic | 14 (33.33) | 31 (27.68) | 45 |  |
| Unknown | 2 (4.76) | 4 (3.57) | 6 |  |
| Currently, my pancreatic cancer is in the following organs |  |  |  |  |
| Liver | 16 (41.03) | 32 (30.48) | 48 | 0.2327 |
| Pancreas | 26 (66.67) | 53 (50.48) | 79 | 0.0827 |
| Peritoneum (abdomen) | 2 (5.13) | 7 (6.67) | 9 | 1.0000* |
| Lung | 2 (5.13) | 10 (9.52) | 12 | 0.5136* |
| Lymph nodes | 10 (25.64) | 11 (10.48) | 21 | **0.0219** |
| None - currently there is no evidence of disease | 6 (15.38) | 35 (33.33) | 41 | **0.0339** |
| I do not know | 1 (2.56) | 5 (4.76) | 6 | 1.0000* |
| Are you currently on treatment, % Yes | 22 (64.71) | 63 (61.76) | 85 | 0.759 |
| Treatment received |  |  |  |  |
| Chemotherapy | 32 (78.05) | 97 (88.18) | 129 | 0.1165 |
| Radiation | 12 (29.27) | 32 (29.09) | 44 | 0.983 |
| Surgery | **16 (39.02)** | **63 (57.27)** | 79 | **0.0459** |
| Enrolled in a clinical trial | 6 (14.63) | 15 (13.64) | 21 | 0.8748 |
| Other treatment(s) not listed above | 3 (7.32) | 10 (9.09) | 13 | 0.7296 |
| Did not receive any treatment | 4 (9.76) | 5 (4.55) | 9 | 0.2549* |
| I am not sure | 2 (4.88) | 0 (0) | 2 | 0.0724* |

*Fisher’s Exact test

**Supplemental Table s3: Patient characteristics by pain NRS.** Patient Information and PC Experience survey items are shown in Column 1. PC patient numbers and (frequencies) of responses to are shown for NRS pain score ranges of NRS 4-8 and NRS 0-3 in Columns 2 and 3, respectively. The total number of responses for both NRS pain score groups is shown in Column 4 and the p-values obtained by comparing the pain group frequency responses per item are shown in Column 5. Percentages represent column percentages for non-missing data related to each item/question.

High NRS 4-8 pain scores reported significantly increased frequencies of earlier age of PC diagnosis, pre-diagnosis Abdominal and/or back pain, Digestive problems and Fatigue. PC patients with Higher pain scores (NRS 4-8) had significantly increased frequencies of “Lymph node” tumor involvement and significantly decreased frequencies of reporting “Yes” to item Currently, there is no evidence of disease, for extent of PC involvement, and item Surgery as part of their PC treatment.

**Supplemental Table s4**

| **Survey time completion** | **High Pain (NRS 4-8) N=26**  **N (%)** | **Low Pain (NRS 0-3)  N=73**  **N (%)** | **Total 99** | **p-value** |
| --- | --- | --- | --- | --- |
| Within 3 months after diagnosis | 11 (37.93) | 18 (62.07) | 29 | 0.0895 |
| >18 months after diagnosis | 15 (21.43) | 55 (78.57) | 70 |  |
|  |  |  |  |  |

**Supplemental Table s4: Time between PC diagnosis to survey completion and % of respondents reporting High vs. Low Pain Rating scores.** A subgroup analysis was conducted based on patients within 3 months of diagnosis (N=29, Column 2) and those greater than 18 months after diagnosis (N=70, Column 3). All other patients completing the survey greater than 3 months and less than 18 months of diagnosis (N=56) were excluded from this analysis. The relationship between the survey time completion variable (3 months vs. >18 months) and the pain intensity variable (High pain NRS 4-8 vs. Low pain NRS 0-3) was assessed by conducting a Chi-square test.

| **Supplemental Table s5. Survey Items** |  | **PC diagnosed**  **prior to 2018 N=1,458 N (%)** | **PC diagnosed year 2018- through 2020 N=514 N (%)** | **Total N=1,972** | **p-value** |
| --- | --- | --- | --- | --- | --- |
| Time in days from diagnosis to survey completion, mean (IQR) |  | 836.39 (127.5-944.5) | 140.0 (24.5-218.0) | 655.90 (69.0-668.0) | **0.0001** |
| Pre-diagnosis A/B Pain, Yes |  | 869 (59.60) | 347 (67.51) | 1,216 | **0.0015** |
| Currently on treatment, % Yes |  | 696 (58.59) | 288 (77.01) | 984 | **0.0001** |
| **Treatment received, % Yes** |  |  |  |  |  |
| Chemotherapy |  | 1142 (82.63) | 351 (72.97) | 1,493 | **0.0001** |
| Did not receive any treatment |  | 99 (7.16) | 78 (16.22) | 177 | **0.0001** |
| Enrolled in a clinical trial |  | 201 (14.54) | 35 (7.28) | 236 | **0.0001** |
| Other treatment(s) not listed above |  | 102 (7.38) | 27 (5.61) | 129 | 0.1885 |
| Radiation |  | 417 (30.17) | 61 (12.68) | 478 | **0.0001** |
| Surgery |  | 723 (52.32) | 138 (28.69) | 861 | **0.0001** |
| I am not sure |  | 3 (0.22) | 2 (0.42) | 5 | 0.6087* |

*Fisher's Exact test

**Supplemental Table s5**: **Treatment options by year of diagnosis.** Variables pertaining to prediagnosis A/B pain and treatment options for PC from PC Experience Survey by year of diagnosis to survey completion status. Percentage values represent column percentages for non-missing data related to each item/question.

Patients diagnosed with PC prior to 2018 vs. those diagnosed in years 2018-2020 report the presence of Pre-diagnosis A/B pain less frequently (59.60 vs. 67.5%, respectively, p=0.0015). The PC patients diagnosed Prior to 2018 vs. between 2018-2020 reported significantly decreased frequencies of item Did not receive any treatment (7.16 vs. 16.22%, respectively, p=0.0001) and significantly increased treatment frequencies of Chemotherapy, Radiation, Surgery and Enrollment in a clinical trial (all p=0.0001). These results may be confounded by shortened time to survey completion and peri-diagnosis evaluation delays while assessing appropriate therapies and clinical trials eligibility due to the shorter time to diagnosis and treatment for many patients.
